# The Successes of the Organised Profession

**Published:** 1920-05-22

**Authors:** 


### THE MATRONS' AND SISTERS' DEPARTMENT.

### THE SUCCESSES OF THE ORGANISED PROFESSION.

It was a bold experiment for the College of Nursing to dispense with an annual subscription from its members and admit them to all its benefits on payment of a guinea fee. This policy has been fully justified by results. The fifth annual report of the College demonstrates that all the main objects for which professional people form associations are being generously fulfilled, and that the finances are in a sound condition. That the public have subscribed to amplify the sphere of work accepted by the College on behalf of nurses by no means implies that the Council of the College has appealed for "charity" in order to benefit their members individually. With admirable perspicuity the Council realised from the beginning that they could neither narrow the scope of their public work nor limit the aid afforded to such indigent members of the profession as had joined the ranks of the College; supported by the British Women's Hospital, they at once invited aid both for endowment purposes and to meet the needs of sick, aged, and infirm nurses. The spirit in which this act of justice towards the whole nursing profession has been started, and the generous way in which givers large and small, and particularly Lord Burnham, through the medium of the *Daily Telegraph*, have lent their support to the Nation's Fund plainly shows the wisdom of this policy.

But though every association is bound to consider the needs of its poorer members and may be judged by its response to their claims, the success of a society of professional persons, such as the College, must be measured by the degree to which it responds to the aspirations of the members for a higher status. In this respect a solid year's work lies to the credit of the College. They have achieved what they set out to do, the State registration of nurses, which hung fire so long and became a workable proposition only when it obtained the support of the entire nursing world, as represented by the College. The educational aims of the College Council have brought new life into the training-schools. The movement for giving university training to sister-tutors and eventually establishing a Chair of Nursing has made great advances during the past year. Eight studentships were awarded, three by the Council of the College, three by an anonymous donor, and two by Lady Cowdray, and the influence exerted through this and other measures in broadening the training of nurses is incalculable.

The College took a great step last year in the publication of the register of members, a costly undertaking well carried through, and a second in the institution of its clever quarterly *Bulletin*, issued at present free of cost to members. No efforts made to increase the interest of members in the parent society are thrown away, and in this connection the issue of the College Badge is a third particularly happy step. Such advances are not made without labour, and have already had an appreciable effect in advancing collective interests.

As regards conferring individual benefits on its members the College has shown great initiative and originality. It dealt with employment difficulties promptly by opening an Appointments and Inquiry Bureau the very week the Armistice was signed, and took a considerable part in the resettlement of the profession after the war. The Bureau is not now needed for this particular purpose, but we are glad to learn it is to be continued as an Information and Inquiry Bureau, for which there is increasing demand not only among members, but among the public.

The free legal advice secured for members through the generosity of Sir Charles Russell has proved a great boon to many, and is the greatest possible safeguard to all. For unscrupulous people will gradually come to understand that a College nurse is protected by the mere fact of her membership from being bullied, and has at her back the strength of the law.

Grants of £15 each were awarded to fifteen members to enable them to take midwifery training, and this good educational branch of aid is to continue and be further developed. It is very satisfactory to learn that a fund will shortly be founded from which loans may be made to members of the College needing assistance to qualify them in any particular branch of nursing or for other purposes.

The public work accomplished by the College is too extensive to be recapitulated here. It has made great demands on those who give their services on Council and Committees, but its effect is far-reaching. Four nurse training-schools have been visited in response to invitations from their respective committees, and in three of these the school has already accepted and carried into effect the recommendations of the Council. We hail with pleasure the announcement in the report that the Council records its support of the Hours of Employment Bill, contingent upon the introduction of modifications suited to the particular needs of the profession. By this they definitely range themselves on the side of a forward policy.

